# Structural and Kinetic Insights Into the Molecular Basis of Salt Tolerance of the Short-Chain Glucose-6-Phosphate Dehydrogenase From *Haloferax volcanii*

**DOI:** 10.3389/fmicb.2021.730429

**Published:** 2021-09-28

**Authors:** Nicolás Fuentes-Ugarte, Sixto M. Herrera, Pablo Maturana, Victor Castro-Fernandez, Victoria Guixé

**Affiliations:** Laboratorio de Bioquímica y Biología Molecular, Departamento de Biología, Facultad de Ciencias, Universidad de Chile, Santiago, Chile

**Keywords:** short-chain dehydrogenase/reductase, glucose-6-phosphate dehydrogenase, archaea, haloadaptation, molecular dynamics simulations

## Abstract

Halophilic enzymes need high salt concentrations for activity and stability and are considered a promising source for biotechnological applications. The model study for haloadaptation has been proteins from the *Halobacteria* class of Archaea, where common structural characteristics have been found. However, the effect of salt on enzyme function and conformational dynamics has been much less explored. Here we report the structural and kinetic characteristics of glucose-6-phosphate dehydrogenase from *Haloferax volcanii* (*Hv*G6PDH) belonging to the short-chain dehydrogenases/reductases (SDR) superfamily. The enzyme was expressed in *Escherichia coli* and successfully solubilized and refolded from inclusion bodies. The enzyme is active in the presence of several salts, though the maximum activity is achieved in the presence of KCl, mainly by an increment in the *k*_*cat*_ value, that correlates with a diminution of its flexibility according to molecular dynamics simulations. The high *K*_*M*_ for glucose-6-phosphate and its promiscuous activity for glucose restrict the use of *Hv*G6PDH as an auxiliary enzyme for the determination of halophilic glucokinase activity. Phylogenetic analysis indicates that SDR-G6PDH enzymes are exclusively present in *Halobacteria*, with *Hv*G6PDH being the only enzyme characterized. Homology modeling and molecular dynamics simulations of *Hv*G6PDH identified a conserved NLTX_2_H motif involved in glucose-6-phosphate interaction at high salt concentrations, whose residues could be crucial for substrate specificity. Structural differences in its conformational dynamics, potentially related to the haloadaptation strategy, were also determined.

## Introduction

The oxidation of glucose-6-phosphate (G6P) to 6-phospho-glucono-1,5-lactone plays an essential role in the oxidative metabolism of glucose, being the first step of the oxidative branch of the pentose phosphate pathway (Fraenkel and Vinopal, 1973). Three different types of non-homologous glucose-6-phosphate dehydrogenases (G6PDH) have been described. The first type corresponds to the well-known G6PDH that belongs to the G6PDH family (Csonka and Fraenkel, 1977; Stincone et al., 2014). These enzymes are tetrameric, with an N-terminal Rossman fold involved in NADP^+^ or NAD^+^ binding and a C-terminal α/β domain where G6P binds (Rowland et al., 1994). This type of G6PDH is widely distributed, being present in bacteria and eukaryotes (Fuentealba et al., 2016; Benítez-Rangel et al., 2020). A second type corresponds to the F_420_-dependent G6PDH, which reduces the F_420_ cofactor required for many F_420_H_2_-dependent reductases present in some methanogens and actinomycetes (Nguyen et al., 2017; Mascotti et al., 2018). These enzymes are homodimers, whose subunit presents an (α/β)8 TIM-barrel fold, with the active site located at the C-terminus of the barrel (Bashiri et al., 2008; Nguyen et al., 2017). Recently, a new type of G6PDH belonging to the short-chain dehydrogenases/reductases (SDR) superfamily has been reported in *Halobacteria* (Pickl and Schönheit, 2015). The enzymes from this superfamily are mainly homodimers that shared a Rossmann-fold of a parallel β-sheet of six to seven β-strands flanked by three to four α-helices from both sides (Kallberg et al., 2010) and include different NAD(P)(H)-dependent oxidoreductases (Kavanagh et al., 2008).

The members of the SDR superfamily are present in the three domains of life, especially in bacteria and eukaryotes. Kallberg et al. (2010) identified 314 SDR families, most of which are present either in Bacteria (178), Eukarya (41), or in both domains (63). In Pickl and Schönheit (2015) reported a new SDR-G6PDH from the halophilic archaeon *Haloferax volcanii* (*Hv*G6PDH). Phylogenetically, the sequence of this enzyme is located inside a putative archaeal G6PDH branch close to L-arabinose dehydrogenases from bacteria and archaea (Pickl and Schönheit, 2015). SDR-G6PDH possesses the Ser/Thr-Tyr-Lys conserved triad, responsible for the common catalytic mechanism proposed for these enzymes (Kallberg et al., 2010), where the Tyr residue acts as a central acid/base catalyst participating in the hydride transfer between the nicotinamide moiety and the sugar. However, for SDR-G6PDH, there is no structural information regarding either its haloadaptation strategy or G6P specificity determinants.

*Haloferax volcanii* belongs to *Halobacteria*, the largest class of the phylum *Euryarchaeota* from Archaea (Larsen, 1967; Mullakhanbhai and Larsen, 1975). Organisms from this class can grow under extreme salinity conditions (3–5 M NaCl) (DasSarma and DasSarma, 2015) and possess an adaptive counterbalance mechanism that allows them to accumulate up to 4 M of K^+^ and Cl^–^ in their cytosol (Youssef et al., 2014). This strategy differs from the one exhibited by some *Methanosarcinales* organisms that accumulate osmolytes, such as glycine betaine, as compatible solutes (Lai et al., 1991). *Methanosarcinales* proteins also present a non-canonical strategy for haloadaptation (Gonzalez-Ordenes et al., 2018), different to the one described for haloarchaeal proteins. The strategy displayed by haloarchaeal proteins comprises mainly three characteristics: (i) increase in the content of negatively charged amino acids, like glutamic and aspartic, (ii) decrease in the content of lysine, and (iii) reduction of the hydrophobic core (Paul et al., 2008; Graziano and Merlino, 2014; Nath, 2016). These structural features are ubiquitously observed in all the haloarchaeal proteins studied to date, and it was considered the canonical strategy for salt adaptation, until the strategy for halophilic *Methanosarcinales* proteins was described. Nonetheless, for *Hv*G6PDH, there is no information regarding the structural characteristics responsible for its haloadaptation strategy or its substrate specificity determinants, especially considering how this specificity appears in the SDR superfamily.

Moreover, halotolerant proteins are very valuable for biotechnological processes that require high salt concentrations and low water activity (Patel and Saraf, 2015). Specifically, G6PDH is commonly used in coupled assays where G6P is measured (Storer and Cornish Bowden, 1974). This reaction is also relevant, for example, in the process that pursue cofactor regeneration from a suitable electron source such as glucose (Zhu et al., 2014) and in the development of electron fuel cells (EFC), like bio-batteries, where electrons are transferred from chemical compounds to electrodes *via* cofactors. In addition, *Hv*G6PDH could be used as an alternative to other G6PDHs in cofactor regeneration under conditions that demanded high salinity in the reaction media.

In this work, we characterized the structural and kinetic features of *Hv*G6PDH. We evaluated the effect of different salts on enzyme function and assessed the ability of *Hv*G6PDH to be employed as an auxiliary enzyme in coupled assays for the determination of glucokinase activity at high salt concentrations. By molecular modeling, we identified putative residues involved in G6P specificity, and by molecular dynamics simulations in the presence and in the absence of salt, we identified structural differences in its conformational dynamics potentially related with the haloadaptation strategy.

Finally, we built a phylogenetic tree with *Hv*G6PDH homologous sequences to analyze its phylogenetic relationships with other SDR enzymes and to determine sequence motifs relevant for substrate specificity in the monophyletic group of SDR-G6PDH.

## Materials and Methods

### Expression, Refolding, and Purification of Recombinant Glucose-6-Phosphate Dehydrogenase From *Haloferax volcanii*

The gene for *Hv*G6PDH (Uniprot code D4GS48), with the optimized sequence for expression in *Escherichia coli*, was synthesized by GenScript USA Inc., (Piscataway, NJ, United States) and subsequently cloned into the vector pET-TEV-28a, which includes a His-tag in the N-terminal and resistance to kanamycin (KAN). The cells of the *E. coli* BL21(DE3) strain were transformed with the expression vector and 50 ml of Luria–Bertani (LB), pH 7.0, supplemented with 30 μg ml^–1^ of KAN and grown overnight with shaking at 200 rpm. Then, 500 ml of the LB/KAN medium was inoculated and supplemented with 1 mM IPTG when the culture reached 0.8–1.2 units of OD_600_ and grown overnight at 37°C. The cells were centrifuged for 10 min at 6,130 *g*, and the pellet was resuspended in 30 ml of lysis buffer (2 M KCl, 50 mM Tris–HCl, pH 7.8, 1 mM EDTA, 5 mM 2-mercaptoethanol, and 0.1% v/v Triton x-114). The resuspended pellet was sonicated with 15 pulses of 20 s at 40% of amplitude, and pauses of 40 s between each pulse were performed. The lysate was centrifuged at 38,360 *g* for 10 min to recover the insoluble fraction corresponding to the inclusion bodies. Afterward, the inclusion bodies were resuspended in 12.5 ml of solubilization buffer (8 M urea, 50 mM Tris–HCl, pH 7.8, 1 mM EDTA, and 50 mM dithiothreitol) and incubated for 1 h at 37°C. After solubilization, the supernatant (solubilized inclusion bodies) was centrifuged at 38,360 *g* for 30 min to recover the soluble fraction. *Hv*G6PDH-solubilized inclusion bodies were filtered through a 0.2-μm syringe filter, refolded by rapid dilution in 237.5 ml of renaturation buffer (2 M KCl, 50 mM Tris–HCl, pH 7.8, and 5 mM 2-mercaptoethanol), and incubated at 4°C overnight with agitation. The refolded inclusion bodies were centrifuged at 38,360 *g* for 20 min, and the supernatant was filtered through a 0.2-μm syringe filter and loaded on a 5-ml HisTrap-HP column (GE Healthcare) equilibrated with renaturation buffer (2 M KCl, 50 mM Tris–HCl, pH 7.8, and 5 mM 2-mercaptoethanol). The column was washed with 10 volumes of buffer A, and protein elution was performed with a linear gradient between 50 and 250 mM imidazole in 50 ml. Fractions with G6PDH activity were pooled, and protein purity was evaluated by sodium dodecyl sulfate polyacrylamide gel electrophoresis (SDS-PAGE). The samples were precipitated with 20% trichloroacetic acid to remove the excess of KCl.

### Kinetic Measurements

Enzyme activity was determined at 25°C by following NADH formation at 340 nm in a buffer of 100 mM Tris–HCl, pH 8.5, 2 M KCl, 3 mM NAD^+^, and 50 mM G6P. A UV/visible Synergy 2 spectrophotometer (BioTek, Winooski, VT, United States) and 96-well plates (model 269620; Nunc, Rochester, NY, United States) were used for measurements. An extinction coefficient of 6.22 mM^–1^ × cm^–1^ for NADH, and a path length correction at 1 cm was used for specific activity (U × mg^–1^) determination. Enzyme unit (U) was defined as micromole of product per minute. To ensure initial velocity conditions, 4–6 nM of protein concentration was used. We determined the kinetic parameters for G6PDH by varying the substrate concentration at a fixed concentration of the co-substrate at pH 7.5 and NaCl and KCl concentrations between 0.5–3.0 and 0.5–2.5 M, respectively. The equation of Michaelis–Menten was adjusted to initial velocities by the non-linear method using the GraphPad Prism 8 software.

The ADP-dependent glucokinase activity was determined using the ADP-dependent GK form *Thermococcus litoralis* (*Tl*GK) by a coupled assay at 25°C following NADH formation at 340 nm in a buffer of 100 mM Hepes, pH 7.5, 3 mM NAD^+^, 5 mM glucose, 1 mM ADP, and 6 mM MgCl_2_. The enzyme concentration was 12 nM, and the KCl concentrations varied between 0.2 and 2.0 M.

### Modeling and Molecular Dynamics Simulation of Glucose-6-Phosphate Dehydrogenase From *Haloferax volcanii*

A homology model of *Hv*G6PDH was constructed using MODELLER, v9.24 (Webb and Sali, 2017). The reported structure of the SDR uronate dehydrogenase from *Agrobacterium fabrum* (PDB: 3RFV) was used as a template since it is the phylogenetically closer protein to HvG6PDH (29.2% identity) with determined three-dimensional structure in the presence of ligands. The 10 models obtained were evaluated based on DOPE score. The model with the best score was used to remodel the non-conserved loop between residues 69 and 80 by *ab initio* modeling using the DaReUS-Loop web tool (Karami et al., 2019), generating 10 loop variants. These models were evaluated using the ProSA-web tools (Wiederstein and Sippl, 2007), Procheck (Laskowski et al., 1993), and Verify3D (Eisenberg et al., 1997) to choose the best one. NAD^+^ and the sugar were added to the best resulting model using MODELLER, v9.24 (Webb and Sali, 2016) software and based on the ligands present in PDB 3RFV. The G6P was built based on the galactaro-1,5-lactone present in the 3RFV PDB structure, changing manually the atomic differences in PyMOL. Finally, the loop β6-α10 (residues from 178 to 190) was re-modeled by the *ab initio* function of MODELLER to solve clashes with G6P.

From the homology model of *Hv*G6PDH, we prepared two systems of molecular simulations, one with 1.5 M of KCl and another without salt. The protonation states were assigned using Propka Software (Olsson et al., 2011) implemented in Maestro, version 9.7 (Schrodinger, LLC, New York, NY, United States). The topology and coordinate input files were prepared using the tLEaP software, and protein parameters were obtained using the ff14SB force field. The parameters for G6P were estimated with Antechamber/Chimera using the GAFF force field, whereas for NAD^+^ the parameters were taken from the Bryce group database (Ryde, 1995). The protein/complex was immersed in a periodic TIP3P water box composed of 25,987 water molecules with 12-Å extension and 58 K^+^ ions to neutralize the charge of the model. For the 1.5 M salt condition, 729 K^+^ ions were added along with the same amount of Cl^–^ counterions.

Once the systems were prepared, a restricted minimization protocol was applied [adapted from Shaw et al. (2010)] with a gradient of spatial restrictions, both to the protein and ligands, to minimize separately the solvent and the protein. These restrictions were gradually released (300, 200, and 10 kcal/mol Å^2^) in three minimization steps, ending with generally 40,000 minimization cycles of the entire system without restrictions. All minimizations were performed in AMBER using pmemd.MPI on a 64-core server. The initial steps utilized the steepest descent method and then switched to a conjugate gradient.

The minimized systems were equilibrated in two steps under the canonical ensemble (NVT: moles, volume, and temperature conserved) conditions; first, for solvent arrangement, the systems were heated to 298 K during 200 ps using a Langevian protocol and 500 kcal/mol Å^2^ restrictions over the protein and ligand atoms. Afterward, the last coordinates of this system were used as a starting point for heating the whole system to 298 K, with no restriction to protein or ligands. This heating was maintained for 1,000 ps, being 75% of this time a ramp from 0 to 298 K.

Then, both systems (low and high salt) were equilibrated under isothermal–isobaric (NPT) ensemble (moles, pressure, and temperature conserved) conditions at a constant temperature of 298 K for 35 ns. From this step, productive trajectories were performed over 100 ns for both conditions, using pmemd.cuda – available in the Amber18 suite, with a time step of 2 fs and periodic border conditions. For each system, three replicas were performed. The energy interactions between the protein and ligands as well as root mean square deviation (RMSD), root mean square fluctuation (RMSF), and radius of gyration (*R*_*g*_) were calculated using CPPTRAJ V4.14.0 (AmberTools V19.12) (Roe and Cheatham, 2013).

To preserve a competent catalytic conformation, a second batch of molecular dynamics, using a protocol like the one described above, was applied. In this case, an extra minimization step was included before the final minimization, and a new set of restrictions (10 kcal/mol Å^2^) to only G6P, NAD^+^, and to the catalytic residue (tyrosine 152) was applied.

A restriction of 10 kcal/mol Å^2^ to ligands and to the catalytic residue Y152 was applied during the second step of the NVT equilibration in both systems. Finally, during the 35 ns NPT equilibration and the 100 ns productive trajectories, a spatial restriction of 1 kcal/mol Å^2^ to G6P and to the hydroxyl group of the Y152 catalytic residue was included.

### Structural Adaptations to High Salt Concentrations

The amino acid distribution in the protein structure was evaluated through the GetArea (Fraczkiewicz and Braun, 1998), and residues were clustered based on solvent accessible surface (SAS). If the SAS is ≥20 Å^2^, the residue is considered exposed to the surface (external shell); if not, it is considered as part of the protein core (internal shell). The electrostatic surface potential was calculated using the APBS tools implemented in PyMOL 2.0 (Baker et al., 2001).

### Inference of Phylogeny of Short-Chain Dehydrogenases/Reductases

We searched for homologous sequences of SDR-G6PDH from *H. volcanii* by a PSI-blast search, with five iterations, in the non-redundant NCBI database and in the UniProtKB database. A non-redundant set with 80% identity was chosen using Multiseq (Roberts et al., 2006), and sequences of characterized SDR members as glucose dehydrogenase (GlcDH) from *E. coli*, human GDP-mannose dehydratase, UDP-glucose-4,6-epimerase from *Arabidopsis thaliana* and *E. coli*, L-arabinose from *H. volcanii*, and uronate dehydrogenase from *A. fabrum* were manually included to identified groups of homolog sequences with an unknown function. A multiple sequence alignment, using ClustalX, was constructed with the initial set of sequences. All these sequences were aligned based on the structural profile of the following structures (PDB ID 3RFV, 3AY3, and 6D9Y). The misaligned positions were manually corrected, and a maximum-likelihood tree was constructed using the PhyML server (Guindon et al., 2010), using the VT evolutive model (Müller and Vingron, 2001) according to AIC-SMS (Lefort et al., 2017).

## Results

### Purification and Kinetic Characterization of Recombinant Glucose-6-Phosphate Dehydrogenase From *Haloferax volcanii*

**FIGURE 1:**
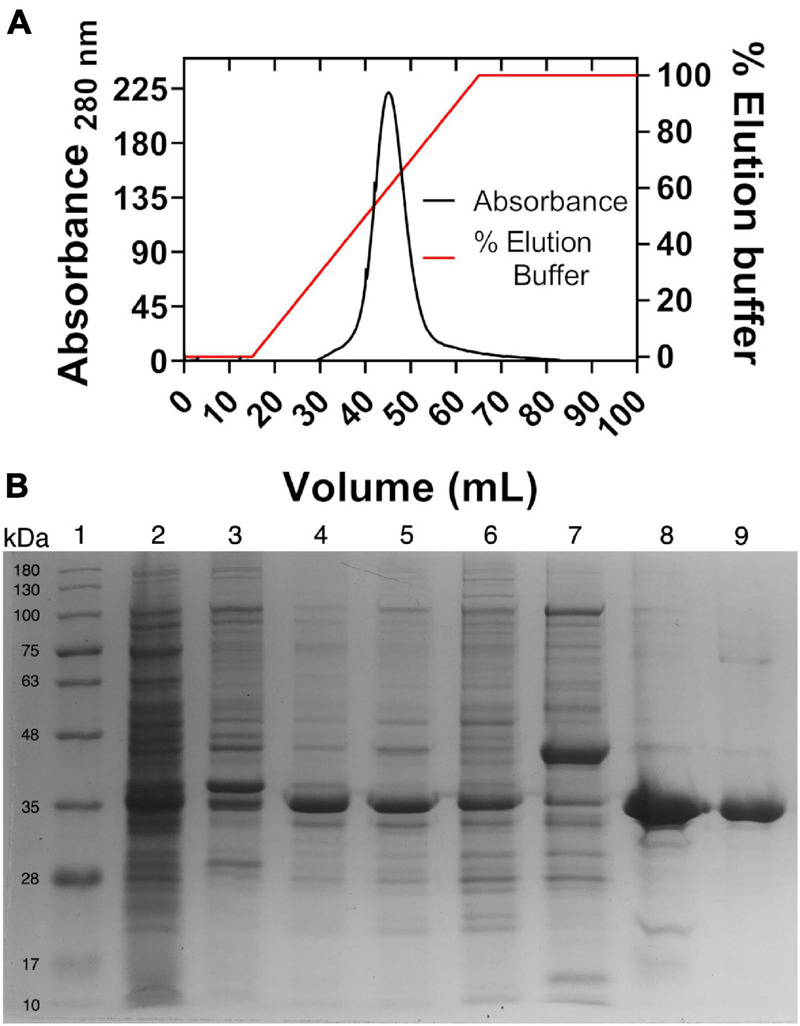
Metal ion affinity chromatography and SDS-PAGE. **(A)** Nickel affinity chromatography. The right axis indicates the percentage of the elution buffer, 100% equal to 250 mM of imidazole. **(B)** SDS-PAGE of purified recombinant glucose-6-phosphate dehydrogenase from *Haloferax volcanii* (*Hv*G6PDH). Lane 1, molecular mass marker; 2, pellet; 3, insoluble fraction; 4, insoluble fraction solubilized with urea; 5, refolded protein; 6, chromatographic front; 7, wash; 8, purified protein precipitated with trichloroacetic acid to remove excess salt; and 9, purified protein after salt remotion by ultrafiltration. Moreover, 10 μg of protein was loaded into each lane.

As was expected, the expression of *Hv*G6PDH in *E. coli* results in inclusion bodies formation, probably due to the requirement of high ionic strength conditions to be properly folded. Therefore, *Hv*G6PDH was purified from inclusion bodies using a refolding protocol as described in the section “Materials and Methods.” The procedure was accomplished with one chromatographic step in a Ni-NTA column, where 5 mg of highly pure *Hv*G6PDH with a specific activity of 134 U/mg was obtained from 500 ml of culture (Figure 1).

We assessed the effect of salt concentration on the optimum pH and kinetic parameters. A significant increase in enzyme activity was observed over 0.5 M of salt, although the optimum pH (between 8 and 10) was not affected by salt (Supplementary Figure 1). The saturation curves for both substrates, G6P and NAD^+^, were determined in the presence of different KCl and NaCl concentrations. In all the conditions tested, the enzyme presents a hyperbolic behavior for both substrates (Supplementary Figure 2). The determination of the kinetics parameters show that an increase in KCl concentrations produced an increment in the *V*_*max*_ value, while the *K*_*M*_ for either NAD^+^ or G6P was not significantly affected (Table 1). A similar effect on the kinetic parameters was observed in the presence of NaCl (Supplementary Figures 2C,D) except for 0.5 M NaCl, where saturation with G6P was not achieved even at 30 mM, and then a high error in the kinetic parameters is expected. The increase in the *k*_*cat*_/*K*_*M*_ values at increasing salt concentrations (either KCl or NaCl) is mainly due to the effect of salt on *k*_*cat*_.

**TABLE 1:** Kinetic constants of glucose-6-phosphate dehydrogenase from *Haloferax volcanii* (HvG6PDH) at 25°C.

	***K*_M_ G6P**	***K*_M_ NAD** ^+^	***k*_cat_ G6P**	***k*_cat_ NAD** ^+^	***k*_cat_*/K*_M_ G6P**	***k*_cat_*/K*_M_ NAD** ^+^
	**(mM)**	**(μM)**	**(s^–1^)**	**(s^–1^)**	**(M^–1^s^–1^)**	**(M^–1^s^–1^)**
**KCl (M)**						
0.5	3.310.51	18544	14.82.4	15.32.6	4.5 × 10^3^	8.3 × 10^4^
1.0	2.330.18	28690	32.41.4	33.03.0	1.4 × 10^4^	1.2 × 10^5^
2.0	2.340.26	28068	49.13.1	48.54.3	2.1 × 10^4^	1.7 × 10^5^
2.5	2.510.33	27326	51.04.5	54.48.4	2.0 × 10^4^	1.9 × 10^5^
**NaCl (M)**						
0.5^*a*^	9.713.39^a^	13066	14.24.7^a^	9.20.9	1.5 × 10^3^	7.1 × 10^4^
1.0	3.470.76	14655	12.91.7	12.50.9	3.7 × 10^3^	8.6 × 10^4^
2.0	2.781.14	243109	21.91.2	21.00.9	7.9 × 10^3^	8.6 × 10^4^
3.0	2.190.42	24583	24.91.5	23.03.7	1.1 × 10^4^	9.4 × 10^4^

*^a^The enzymatic activity was assayed up to 30 mM G6P, but saturation was not reached. The mean ± SD was calculated from three independent measurements.*

**FIGURE 2:**
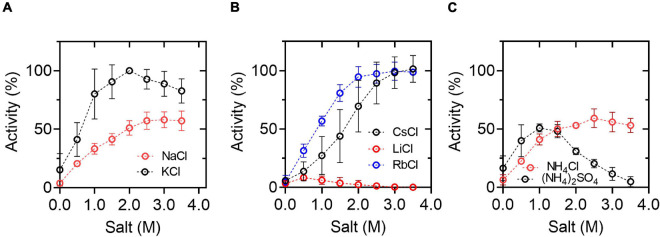
Effect of chloride salts on *Hv*G6PDH activity. **(A)** Activity curves in the presence of KCl or NaCl. **(B)** Activity curves in the presence of CsCl, LiCl, or RbCl. **(C)** Activity curves in the presence of NH_4_Cl or (NH_4_)_2_SO_4_. The activity was assayed at 25°C in the presence of 30 mM G6P and 3 mM NAD^+^. In all assays, 100 mM of residual KCl was present due to the *Hv*G6PDH storage buffer. The data was normalized, taking the activity that registered at 2.0 M KCl as 100%. The means and standard deviations of three replicates are shown.

To explore the biotechnological potential of this halophilic enzyme, we assess the effect of different salts on the enzyme activity under saturating substrate conditions (considering the parameters obtained for NaCl and KCl) (Figure 2). In agreement with the halophilic character of the enzyme, maximum activity was achieved in the presence of KCl or NaCl (over 1.0 M of KCl or NaCl), although the activity is 1.9-fold higher in the presence of 2 M KCl compared to the same NaCl concentration. Other salts, like CsCl, NH_4_Cl, and RbCl, also produce an increment in enzyme activity, although the maximum activity is lower than the one obtained in the presence of KCl. On the other hand, LiCl and, to a lesser extent, (NH_4_)_2_SO_4_ produced an inhibitory effect.

Since G6PDH is an enzyme usually employed in coupled assays to measure glucose phosphorylation reactions, we explore the ability of *Hv*G6PDH to be used as an auxiliary enzyme for halophilic hexokinase/glucokinase reactions. As a control, it is necessary to determine the ability of *Hv*G6PDH to oxidize glucose in the presence of NAD^+^ (GlcDH activity). This was assessed by performing glucose saturation curves in the presence of KCl or NaCl (Figure 2). In the presence of NAD^+^, *Hv*G6PDH oxidizes glucose with a linear behavior even up to 500 mM. The catalytic efficiency (*k*_*cat*_/*K*_*M*_) of the GlcDH activity in the presence of KCl and NaCl is 2.3 × 10 and 1.7 × 10^1^ M^–1^s^–1^, respectively, values that are more than two orders of magnitude lower than the ones obtained for the G6PDH activity (Table 1).

**FIGURE 3:**
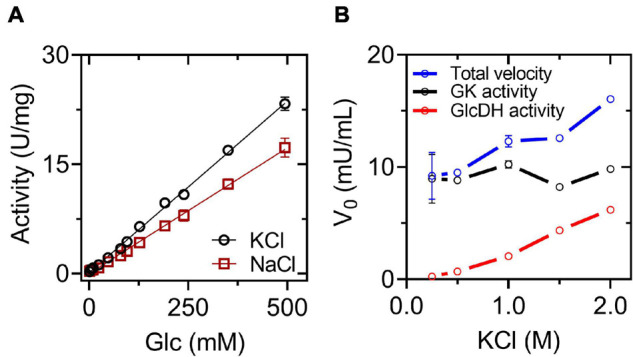
Glucose dehydrogenase (GlcDH) activity and coupled assay for GK activity. **(A)** Saturation curves for glucose at 2 M salt. The reactions were assayed at 25°C in the presence of 3 mM NAD^+^. **(B)** Coupled assay for GK form *Thermococcus litoralis* (*Tl*GK), employing 2 U/ml of *Hv*G6PDH as an auxiliary enzyme at 25°C in the presence of 3 mM NAD^+^, 5 mM glucose, 1 mM ADP, and 6 mM MgCl_2_. The means and standard deviations are shown. For **(A)**, *n* = 3, and for **(B)**, *n* = 2.

Based on the mathematical approximation reported by Storer and Cornish-Bowden (Storer and Cornish Bowden, 1974), we design a glucokinase (GK)-coupled assay by employing *Hv*G6PDH as an auxiliary enzyme, considering its GlcDH activity. We calculated the minimum amount of *Hv*G6PDH required to measure an initial velocity of 0.01 mM min^–1^ for the GK activity, with a lag time of 1–5 min before the steady state is reached and with a *v*_2_/*v*_1_ ratio of 0.95–0.99 [the *v*_2_/*v*_1_ ratio corresponds to the quotient between the auxiliary enzyme velocity (*v*_2_) and the interest enzyme velocity (*v*_1_) at the steady state] (Supplementary Table 1). Considering these parameters, 2 U/ml of *Hv*G6PDH should be used. This was experimentally tested with the hyperthermophilic ADP-dependent glucokinase from *T. litoralis*. First, we measured the GlcDH activity in the presence of increasing concentrations of KCl and saturating concentrations of NAD^+^ and glucose. Then, *Tl*GK addition causes a significant increase in the initial velocity, which allows us to calculate the actual glucokinase activity by subtracting the GlcDH velocity (Figure 3). However, the GlcDH activity displayed by *Hv*G6PDH restricts its use as an auxiliary enzyme for GK-coupled assays to conditions where low glucose concentrations are employed (below 1 mM); otherwise, a careful determination of the GlcDH activity must be performed. Despite these limitations, the *Tl*GK hyperthermophilic enzyme remains active up to 2.0 M of KCl, indicating that *Hv*G6PDH can be used as an auxiliary enzyme with the appropriate controls.

### Molecular Modeling of Glucose-6-Phosphate Dehydrogenase From *Haloferax volcanii* and Glucose-6-Phosphate Binding Site

**FIGURE 4:**
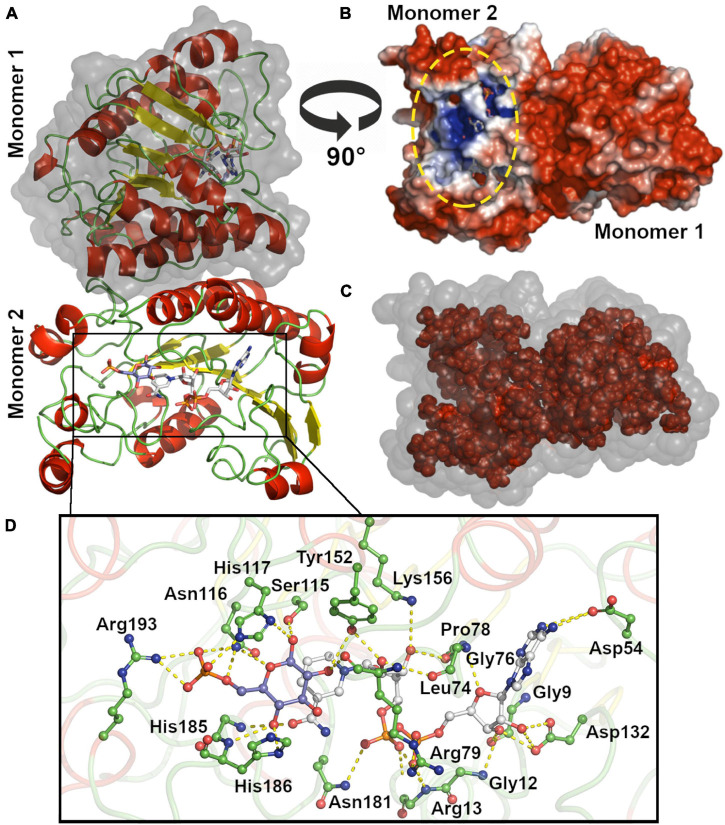
Molecular modeling and active site of *Hv*G6PDH. **(A)** Dimeric *Hv*G6PDH architecture. Each monomer is represented as a cartoon: α helix (red), β-sheets (yellow), and loops (green). The surface of one of the monomers is shown in gray. **(B)** Surface electrostatic potential for the *Hv*G6PDH model, being blue for positive and red for negative potential (± 3 kB T/e). The structure is represented as the solvent-excluded surface (Connolly surface). The yellow oval denotes the active site. **(C)** Residues from the hydrophobic core (inner shell), using as criteria less than 20 Å^2^ exposed. The structure is represented as the solvent accessible surface (SAS); the surface is represented in gray and the residues from the inner shell as red spheres. **(D)** Active site residues. The residues at 5 Å from G6P and NAD^+^ ligands are shown, and those establishing polar interactions are represented as sticks and balls. The carbon atoms of the G6P molecule are shown in blue–purple, those of NAD^+^ in white, and the carbon of the amino acid residues in green. The oxygen atoms are red and nitrogen blue.

We modeled the dimeric structure of *Hv*G6PDH (Figure 4A) using the non-halophilic uronate dehydrogenase from *Agrobacterium fabrum* (*Af*UDH, PDB ID: 3RFV) as a template (Figure 4A). The *Af*UDH is the protein phylogenetically closer to HvG6PDH (29.2% identity) with a determined structure in the presence of ligands. The region between residues 69–80 of *Hv*G6PDH was *ab initio* modeled using DaReUs-loop (Karami et al., 2019) since it lacks any homology with the template. Moreover, when ligands (G6P and NAD^+^) were added to the model, significant clashes between them and the residues located in the 178–193 region (β6-α10 loop) were found. For this reason, this loop was also *ab initio* modeled using the loop refine tool of MODELLER. After modeling, we selected the model with the best DOPE score and the higher number of interactions between the model and G6P. The selected model was minimized with ff14SB force field in Amber18. For this minimized structure, the surface electrostatic potential and the solvent accessibility surface of both monomers were calculated. For comparison, these parameters were also calculated for the non-halophilic *Af*UDH. For *Hv*G6PD, a markedly negative surface potential was observed over the entire protein (Figure 4B), in contrast with the non-halophilic *Af*UDH (Supplementary Figure 3B). The results obtained agree with those described for haloarchaeal proteins (Britton et al., 2006; Baker et al., 2009) where a high presence of negatively charged residues, such as aspartic and glutamic, are observed (Supplementary Figure 4). However, in both the halophilic as well as the non-halophilic protein, a region of positive electrostatic potential is observed, which corresponds to the substrate binding site, according to the chemical nature of NAD^+^ and G6P, which have bulky negatively charged groups.

The hydrophobic core of *Hv*G6PDH and *Af*UDH is mainly composed of small hydrophobic residues, like alanine, glycine, leucine, and valine (Figure 4C and Supplementary Figure 3C). Nevertheless, *Hv*G6PDH shows an important diminution of leucine (7.9% *Hv*G6PDH and 14.9% *Af*UDH) and a slight increase in alanine content (13.4% *Hv*G6PDH and 10.8% *Af*UDH) when compared to the non-halophilic *Af*UDH. Besides this, most of the tryptophan residues of the halophilic *Hv*G6PDH are exposed to the solvent (five out of six are exposed), unlike the non-halophilic protein where most of them are located at the hydrophobic core (only 2 out of 10 are exposed). The halophilic protein also presents a reduction in the hydrophobic core, which represents 39.4% of the total volume of the protein, in comparison with the non-halophilic protein where the hydrophobic core represents 45.9%. These results agree with the structural adaptations reported for haloarchaeal proteins (Paul et al., 2008; Graziano and Merlino, 2014).

The minimized model of *Hv*G6PDH shows that the binding of G6P and NAD^+^ at the active site involves residues from the β6-α10 loop in the first coordination shell. Residues H186 and R193 establish polar interactions with G6P, whereas N181 interacts with the di-phosphate of the NAD^+^ molecule and residue H185 with the nicotinamide moiety. The other residues participating in G6P stabilization are N116 and H117 (Figure 4D). Residues located at 5Å from NAD^+^ are mainly glycine and aspartic that interact with the adenosine moiety and residues R13 and R79 that interact with the di-phosphate group. The catalytic residues reported for SDR superfamily members are also found at the modeled active site of *Hv*G6PDH, and correspond to S115, Y152, and K156. These results indicate that *Hv*G6PDH would perform its catalytic function through the conserved mechanism described for SDR superfamily members, and this allows us to propose a role of the aforementioned residues in substrate binding.

### Molecular Dynamic of Glucose-6-Phosphate Dehydrogenase From *Haloferax volcanii* at Low and High Salt Concentrations

**FIGURE 5:**
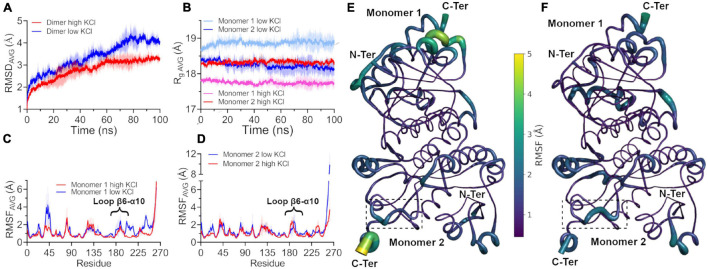
Conformational dynamics of *Hv*G6PDH at different salt concentrations. The colored lines show the average root mean square deviation (RMSD) values of three 100-ns replicates without spatial restriction on ligands; ± SD is shown as a shaded area. **(A)** Average RMSD values of the *Hv*G6PDH dimer in the presence of low salt (blue) and high salt (red) from classical molecular dynamics. **(B)** Average radius of gyration (*R*_*g*_) of each *Hv*G6PDH monomer. The colors with red hues represent the simulation with high salt, and the blue ones represent simulations in the presence of low salt. Monomer 1 is represented by pink and light blue and monomer 2 by red and blue. **(C,D)** Average root mean square fluctuation (RMSF) values for monomers 1 and 2, respectively, at both high and low salt concentrations. Both monomers exhibit regions with high RMSF values, such as the β6-α10 loop. **(C)** Particularly, in the case of monomer 1, these regions display higher RMSF values at low salt but are stabilized at high salt concentrations. **(E,F)** Putty structural representation of the *Hv*G6PDH dimer with the average RMSF values depicted as a color scale for low and high salt molecular dynamics, respectively. The β6-α10 loop of monomer 2 is displayed by a dotted square.

To assess the effect of molar concentrations of salt on the structural dynamics of the dimeric structure of *Hv*G6PDH, we performed classical molecular dynamics simulation (cMDS) at a constant temperature of 300 K for 100 ns at low salt concentration (58 atoms or 125 mM of K^+^), only providing the necessary counter-ions to maintain electrolytic neutrality in the system. A second batch of cMDS was also performed, where K^+^ and Cl^–^ ions (729 atoms of each) were added to simulate a 1.5-molar concentration of salt. The RMSD values are shown to evaluate the conformational integrity and the global changes of the *Hv*G6PDH model at low and high salt concentrations. The average (±SD between replicates) RMSD values of three replicates at low KCl reached 4.08 ± 0.19 Å in the last 10 ns, whereas at 1.5 M KCl, this value is only 3.30 ± 0.14 Å (Figure 5A). To determine the basis of this difference, we separately analyzed the *R*_*g*_ of each monomer of the protein in both conditions (low and high salt). The average value during the trajectory is different for both monomers; in the case of monomer 1, *R*_*g*_ is 17.8 Å at high and 18.8 Å at low KCl concentration. Nonetheless, monomer 2 maintains a very similar *R*_*g*_ average in both conditions over all the trajectories (18.3 Å at high KCl and 18.2 Å at low KCl) (Figure 5B). This result suggests a role for salt concentration in the conformational dynamics of *Hv*G6PDH; at low salt concentration, one of the monomers is more swollen, which could be the result of partial unfolding of the protein or a higher conformational dynamic. Then, we analyzed the atomic fluctuation of our model, evaluating each monomer as an independent chain. At low KCl concentration, the overall mean RMSF is 1.36 ± 0.30 Å (monomer 1) and 1.32 ± 0.28 Å (monomer 2). In contrast, at high KCl concentration, the RMSF values are lower than those obtained at low salt, 1.03 ± 0.19 and 1.08 ± 0.19 Å for monomers 1 and 2, respectively. A more detailed analysis of each monomer in both conditions revealed regions with high RMSF variations in monomer 1 (Figures 5C,D). At low salt concentration, regions such as the α2 helix, the β6-α10 loop, and the segment from α11 helix to β7 sheet of monomer 1 present very high RMSF average values, with these regions being stabilized at high salt concentration (Figures 5E,F).

Moreover, a close inspection of the substrates located at the active site shows that they do not maintain a catalytic conformation in replicates of either condition. To analyze the enzyme–substrate interactions, a 1-kcal/mol Å^2^ restriction to G6P, NAD^+^, and the catalytic tyrosine (Y152) was applied, and a new set (3) of molecular dynamics was performed at low and high salt concentrations. In these new systems, the substrates maintain a catalytic conformation, and the potential energy of non-bonded interactions as well as the electrostatic and the van der Waals component between G6P and the protein were evaluated. The average values for the electrostatic interactions at low KCl were −196.09 ± 21.84 and −230.77 ± 14.54 kcal/mol Å^2^ for monomers 1 and 2, respectively. On the other hand, these values for the van der Waals interactions were −26.37 ± 3.45 kcal/mol Å^2^ for monomer 1 and −15.95 ± 2.86 kcal/mol Å^2^ for monomer 2 (Supplementary Figure 5A). These results indicate that attractive electrostatic interactions are the main energetic component in the interaction between G6P and the protein. At high KCl concentration, the average value for the electrostatic component in monomers 1 and 2 are −192.85 ± 51.73 and −114.50 ± 35.20 kcal/mol Å^2^, while for the van der Waals interactions these are −16.29 ± 3.13 and −19.45 ± 3.49 kcal/mol Å^2^, respectively (Supplementary Figure 5B). The variation observed in the mean values of the electrostatic component could be attributed to a shielding effect due to the high ion concentration, although the electrostatic interactions of the protein with G6P were still attractive.

**FIGURE 6:**
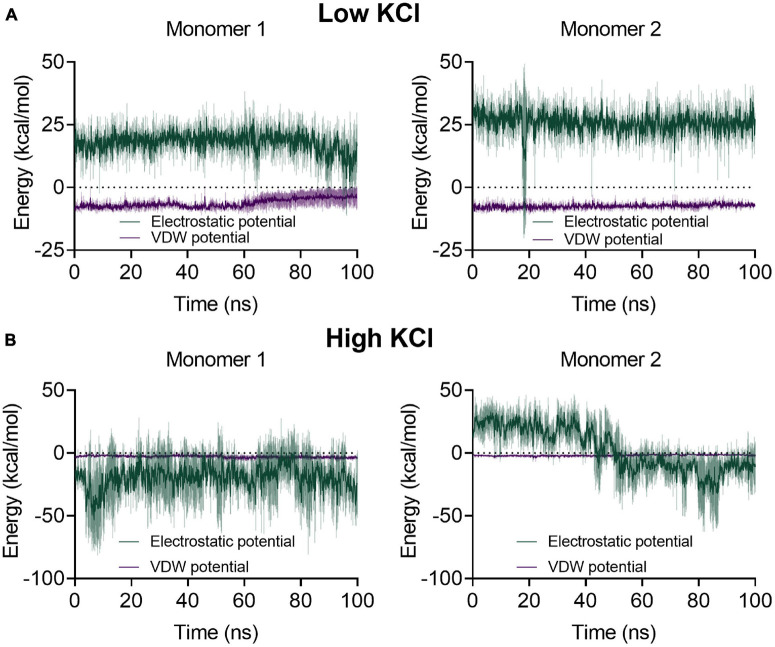
Average electrostatic and van der Waals potential energies between the β6-α10 loop and glucose-6-phosphate (G6P) for each monomer at low and high salt concentrations. The colored lines represent the average values of three 100-ns replicates with a 1-kcal/mol Å^2^ restriction to G6P and to the hydroxyl group of the catalytic residue Y152; the ± SD is represented as a shaded area. **(A)** At low salt concentration, the energy profile of both monomers displays an electrostatic repulsion between the loop and G6P and a lower attractive interaction for the Van der Waals potential. **(B)** During the high salt molecular dynamics, monomer 1 displays an electrostatic attraction between the loop and G6P through most of the dynamic, whereas the loop in monomer 2 begins with an average electrostatic repulsion and then changes to an average attractive interaction.

Interestingly, the β6-α10 loop, which was modeled *ab initio*, shows a significant change in the molecular trajectories in the presence of low or high salt. At low salt concentration, the electrostatic component of the interactions between this loop and G6P is mainly repulsive, being +17.90 ± 5.05 and +25.74 ± 5.16 kcal/mol Å^2^ for monomers 1 and 2, respectively (Figure 6). Surprisingly, in all the replicates performed at high salt concentrations, the electrostatic component became attractive, being −19.58 ± 17.19 kcal/mol Å^2^ for the first monomer. However, for the second monomer, the average value diminished from 22.49 ± 4.92 in the first 25 ns of the simulation to −14.92 ± 7.66 in the last 25 ns, changing from a repulsive to an attractive interaction (Figure 6).

### Phylogenetic Analysis of Glucose-6-Phosphate Dehydrogenases From *Haloferax volcanii*

**FIGURE 7:**
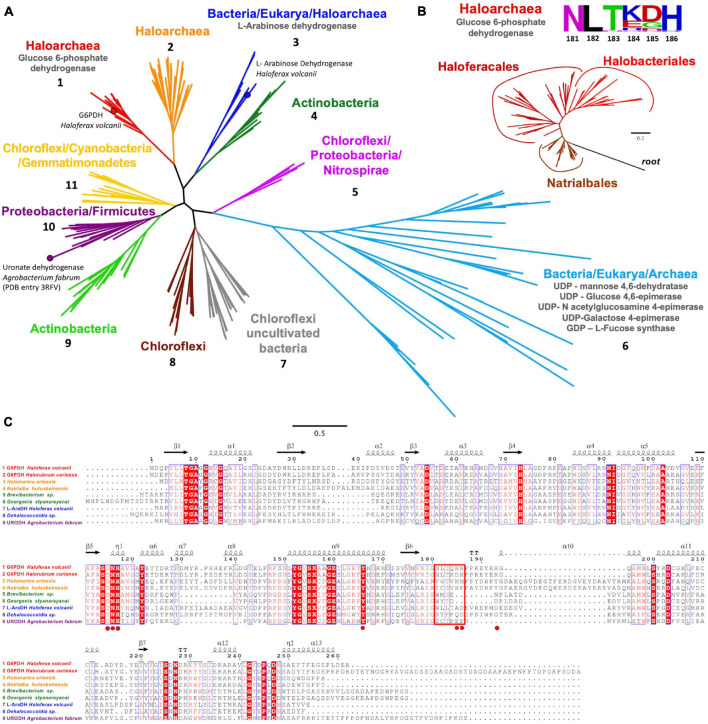
Phylogenetic relationship and sequence analysis of *Hv*G6PDH. **(A)** Monophyletic branches (numbered from 1 to 11) with their respective functions are represented in different colors. **(B)** Phylogenetic tree of glucose 6-phosphate dehydrogenase from *Halobacteria* showing the segregating branches of *Natrialbales*, *Halobacteriales*, and *Haloferacales*. Sequences from the short-chain dehydrogenases/reductases (SDR) superfamily were used as the outgroup. The sequence logo for the β6-α10 loop motif is also shown. **(C)** Sequence alignment of representative enzymes from groups one to four and the uronate dehydrogenase from *Agrobacterium fabrum*. The red rectangle indicates the β6-α10 loop motif. The G6P interacting residues for *Hv*G6PDH are highlighted in red circles. The protein sequence codes used in the phylogenetic inference are listed in Supplementary Table 3.

The evolutionary relationship of archaeal *H. volcanii* G6PDH with SDR superfamily members was analyzed by molecular phylogeny using a PSI-Blast search. Sequences of characterized SDR enzymes, like UDP-glucose 4,6 epimerase, GDP-L-fucose synthase, L-arabinose dehydrogenase, and uronate dehydrogenases from eukaryotes, bacterial, and archaeal lineages, were included in the alignment. We identified 11 monophyletic groups (Figure 7A) where *Hv*G6PDH appears in a group of uncharacterized sequences from *Halobacteria* organisms, including the orders *Natrialbales*, *Haloferacales*, and *Halobacteriales* (Figure 7A, group 1, and Figure 7B). Members of this group have the conserved NLTX_2_H motif of the β6-α10 loop (sequence logo in Figure 7B) identified as relevant for the interaction with G6P in molecular dynamic simulations. A second conserved motif of this group also corresponds to the YERG motif, where R193 could be important for phosphate binding (Figure 7C). Close to group 1, another uncharacterized group of *Halobacteria* sequences appear (Figure 7A, group 2), whose sequences show an insertion inside the β6-α10 loop, which could indicate a different substrate specificity (Figure 7C). A branch composed exclusively of bacterial sequences including *Chloroflexi*, *Proteobacteria*, and *Nitrospira* organisms (group 5) was also identified. The most populated group (group 6) comprises enzymes with activities like dehydrogenases, epimerase–dehydratase, and fucose synthase from the three domains of life. The other four groups are composed exclusively by sequences from different lineages of bacteria with unknown enzymatic function, except for group 10, where the sequence of uronate dehydrogenase from *A. fabrum* is located.

## Discussion

The use of *E. coli* as host for recombinant haloarchaeal protein production has been challenging, probably due to the requirement of these proteins of molar concentrations of ions to fold properly. Since in most of the cases the studied proteins are obtained as inclusion bodies (Martínez-Espinosa, 2020), several protocols have been described to solubilize and refold different proteins (Connaris et al., 1999; Camacho et al., 2002; Díaz et al., 2006). This was the case for the expression of *Hv*G6PDH in *E. coli*, where we used a solubilization and refolding protocol, similar to the one described by Pire (Pire et al., 2001), that enabled us to obtain the purified enzyme in a soluble and active form, with kinetic parameters comparable to those reported by Pickl and Schönheit (2015), who characterized the enzyme obtained from the original organism (*H. volcanii*).

Although the haloadaptation structural properties of proteins have been extensively studied, with all of them showing a common strategy, the effect of salt on the kinetics parameters of these enzymes varies widely. To date, halophilic enzymes from *Halobacteria* organisms show either a diminution or increase in the *K*_*M*_ for substrates (Blecher et al., 1993; Madern and Zaccai, 2004), an increase in *k*_*cat*_ (Baxter, 1959; Lanyi, 1974), or a diminution in *K*_*M*_ and an increase in *k*_*cat*_ (Souza et al., 2012) at high salt concentrations. Moreover, for these proteins, different salt preferences have been reported (Lanyi, 1974; Prüß et al., 1993; Wright et al., 2002) – for example, the isocitrate dehydrogenase from *Halobacterium salinarum* displays its maximum activity in the presence of NaCl (McClure, 1969), while for glyceraldehyde-3-P dehydrogenase from *Haloarcula vallismortis* it was obtained in the presence of RbCl (Krishnan and Altekar, 1990). On the other hand, the dihydrofolate reductase from *H. volcanii* shows its maximum activity in the presence of KCl (Wright et al., 2002), as was observed here for the G6PDH from the same organism. In the case of *Hv*G6PDH, either NaCl or KCl exerts their action through an increment in *k*_*cat*_, whereas the *K*_*M*_ for both substrates was not significantly affected. The maximum activity of *Hv*G6PDH achieved in the presence of KCl would reflect a physiological adaptation since it has been reported that the KCl concentration in the cytoplasm of *H. volcanii* is around 3.4 M (Pérez-Fillol and Rodríguez-Valera, 1986). The effect of different salts on the stability and activity of enzymes from the *Halobacteria* class has also been reported, focusing on properties like the ionic radii, ionic force, density charge, crowding effect, exclusion volume, and Hofmeister effect (Broering and Bommarius, 2005).

Our results suggest that *Hv*G6PDH requires a particular ionic radii and density charge to achieve its maximum activity, with physiological ion potassium being the most effective. At a density charge higher or lower compared to K^+^, the enzyme activity diminishes, which is the major effect observed with ions like Li^+^ and Na^+^ that have the highest density charges and then less ionic radii. This can also be interpreted regarding the protein stabilizing effect of the Hofmeister series cations that follow the NH_4_^+^, K^+^, Na^+^, Cs^+^, and Li^+^ order (Patel et al., 2014). Cations with high density charge, like Mg^+2^, Ca^+2^, and Li^+^, tend to stabilize the protein when they are present at low concentrations, while at higher concentrations they denature the protein, which could explain the absence of *Hv*G6PDH activity in the presence of LiCl.

The effect of KCl on the kinetic parameters of *Hv*G6PDH could be due to a diminution of its flexibility since MDS shows a compaction of the structure (*R*_*g*_) and a lower RMSF average value at high salt concentration. These results agree with what is observed in the β-galactosidase from extremophiles, where the halophilic enzymes display a higher flexibility in the absence of salt compared to its mesophilic and thermophilic homologs (Karan et al., 2020).

Until recently, it was generally assumed that the oxidative branch of the pentose-phosphate pathway was absent in most of the archaeal organisms studied since homologs of classical bacterial G6PDH could not be identified in any archaeon, and a modified version of this pathway was proposed (Soderberg, 2005). Nevertheless, to date, the presence of G6PDH as well as 6-phosphogluconate dehydrogenase has been reported in *Haloabacteria*, *Pacearchaeota*, *Thaumarchaeota*, and *Asgarchaeota* organisms (Maturana et al., 2021), although SDR-G6PDH seems to be restricted to *Halobacteria*, as has been shown previously by Pickl and Schönheit (2015).

Glucose-6-phosphate dehydrogenase from *Haloferax volcanii* conserves the catalytic triad (S115, Y152, and K156) of the SDR superfamily, where the hydride transfer mechanism would be mediated by the tyrosine residue and the lysine residue that establishes hydrogen bonding with the ribose moiety of NAD^+^ and the serine residue that would interact with the reaction intermediary. On the other hand, for canonical G6PDH, a different reaction mechanism has been described, where the electron transfer is carried out by a histidine residue (Cosgrove et al., 1998), indicating a case of convergent evolution of function.

Phylogenetic analysis indicates that SDR-G6PDH is present only in *Halobacteria*, with G6PDH from *H. volcanii* being the only enzyme characterized from this group. Unlike canonical G6PDH present in Bacteria and Eukarya, *Hv*G6PDH is preferred by NAD^+^ and displays a promiscuous activity toward glucose as substrate, which restricts its use as an auxiliary enzyme. Employing mathematical models to determine the minimal units of G6PDH to be used in a couple assay to measure glucokinase activity, it was evidenced that the *K*_*M*_ for G6P of the G6PDH auxiliary enzyme is a critical factor (McClure, 1969; Storer and Cornish Bowden, 1974). To this respect, it is worth noting that *Hv*G6PDH has a *K*_*M*_ for G6P in the millimolar range, while for canonical non-homologous G6PDH enzymes these values are in the micromolar range (Cosgrove et al., 1998; Ma et al., 1998; Kotaka et al., 2005). This fact, along with *Hv*G6PDH glucose promiscuous activity, imposes restrictions to its use as auxiliary enzyme, such as low glucose concentration (1 mM or less) and less than 2 U/ml units of *Hv*G6PDH, to avoid long lag times. Even so, in the absence of other halophilic G6PDH, *Hv*G6PDH represents a valuable starting point for its use as a scaffold to be engineered for either diminishing its *K*_*M*_ for G6P, abolishing its GlcDH activity, or both.

In this respect, molecular dynamics simulations of *Hv*G6PDH in the presence of substrates provide relevant information of G6P specificity structural determinants. The NLTX_2_H motif of the β6-α10 loop exhibits attractive interactions with G6P at high salt concentrations, where H186 may be crucial for sugar phosphate interaction and then for G6P over glucose specificity. Other residues, like R193 and H117, have been identified as important for their interaction with the phosphate group of the substrate. However, further studies will be needed to experimentally determine the role of these residues in G6P specificity in this new G6PDH enzyme from the SDR superfamily.

## Data Availability Statement

The raw data supporting the conclusions of this article will be made available by the authors, without undue reservation.

## Author Contributions

NF-U contributed to the conceptualization, investigation (designed and carried out protein purification protocol, kinetics experiments, and molecular modeling), data curation, performed the formal analysis, and wrote the original draft. SMH contributed to the investigation (designed and carried out molecular dynamics simulation), data curation, performed the formal analysis, and wrote the original draft. PM contributed to the investigation (designed and carried out molecular phylogeny), data curation, performed the formal analysis, and wrote the original draft. VC-F contributed to the conceptualization, performed the formal analysis, supervised the study (protein purification, kinetics experiments, molecular modeling, and molecular dynamics simulation), and reviewed and edited the original draft. VG contributed to the conceptualization, performed the formal analysis, supervised the study (protein purification, kinetics experiments, and molecular phylogeny), took charge of funding acquisition and project administration, and reviewed and edited the original draft. All authors discussed the results and commented on the manuscript.

## Conflict of Interest

The authors declare that the research was conducted in the absence of any commercial or financial relationships that could be construed as a potential conflict of interest.

## Publisher’s Note

All claims expressed in this article are solely those of the authors and do not necessarily represent those of their affiliated organizations, or those of the publisher, the editors and the reviewers. Any product that may be evaluated in this article, or claim that may be made by its manufacturer, is not guaranteed or endorsed by the publisher.
