# Supplementary material for: Structural and Kinetic Insights Into the Molecular Basis of Salt Tolerance of the Short-Chain Glucose-6-Phosphate Dehydrogenase From Haloferax volcanii
Source: Front Microbiol. 2021 Sep 28;12:730429. doi: 10.3389/fmicb.2021.730429 (PMC8506132; doi:10.3389/fmicb.2021.730429)
Supplement: Supplementary file 1 [file Data_Sheet_1.PDF]

Supplementary Material

Supplementary Tables

Supplementary Table S1. Estimation of auxiliary enzyme units to be used in the coupled assay.

v_2/v_1[1]	t^* *(min)*[2]	φ[3]	v_1/V_2[4]	V_2 *(U/ml)*[5]
**0.99**	1.0	$3.06\cdot10^{-3}$	$6.60\cdot10^{-4}$	15.2
**0.99**	2.0	$6.12\cdot10^{-3}$	$1.33\cdot10^{-3}$	7.5
**0.99**	3.0	$9.17\cdot10^{-3}$	$1.99\cdot10^{-3}$	5.0
**0.99**	4.0	$1.22\cdot10^{-2}$	$2.65\cdot10^{-3}$	3.8
**0.99**	5.0	$1.53\cdot10^{-2}$	$3.32\cdot10^{-3}$	3.0
**0.95**	1.0	$3.06\cdot10^{-3}$	$1.02\cdot10^{-3}$	9.8
**0.95**	2.0	$6.12\cdot10^{-3}$	$2.04\cdot10^{-3}$	4.9
**0.95**	3.0	$9.17\cdot10^{-3}$	$3.06\cdot10^{-3}$	3.3
**0.95**	4.0	$1.22\cdot10^{-2}$	$4.07\cdot10^{-3}$	2.5
**0.95**	5.0	$1.53\cdot10^{-2}$	$5.11\cdot10^{-3}$	2.0

[1] v_2/v_1 is the ratio between the coupled reaction velocity (v_2) and the interest reaction velocity (v_1).

[2] t* is the time required to reach the steady state.

[3] φ is a is a dimensionless number and a function of the ratios v_2/v_1 and v_1/V_2.

[4] v_1/V_2 is the ratio between v_1 and V_2.

[5] V_2 is the amount of auxiliary enzyme in U/mL.

Supplementary Table S2. Structural characteristics of halophilic *Hv*G6PDH and non-halophilic *Af*UDH.

	Inner shell volume (Å ^{3})	Outer shell volume (Å ^{3})	Percentage inner shell	Charge at pH 7.0
***Hv*G6PDH**	27,174	41,758	39.4	-38
***Af*UDH**	31,857	37,485	45,.9	-10

Supplementary table S3. Uniprot or NCBI reference sequence codes used in the phylogenetic tree.

AAC50786.1	WP_142979896.1	PSP76558.1	MQA27186.1	WP_004041122.1
ACX98429.1	ESS07378.1	WP_128223295.1	WP_116709618.1	MBS11416.1
WP_011305081.1	WP_089764659.1	WP_058583356.1	WP_068689561.1	MBK34963.1
WP_022441977.1	WP_004044412.1	WP_103428312.1	WP_076440938.1	RIK09838.1
ADE03524.1	CDK39514.1	WP_089815714.1	WP_133900628.1	WP_141337963.1
Q14376.2	WP_006111491.1	WP_050460454.1	WP_159697925.1	WP_094452143.1
ETW61934.1	WP_137717009.1	WP_124955173.1	WP_132124463.1	WP_160953240.1
EMH75851.1	WP_137688129.1	ESP87066.1	WP_112263417.1	WP_153573112.1
WP_000659810.1	WP_088901668.1	WP_160061642.1	WP_123743337.1	WP_137801913.1
WP_011032610.1	WP_092567269.1	WP_049903763.1	WP_121221652.1	WP_034401108.1
Q8T8E9	WP_144800044.1	SEP25550.1	MBD06212.1	WP_017824009.1
ADE04757.1	WP_094527679.1	WP_087715628.1	NBY49079.1	WP_076061474.1
A3MUJ4	WP_127116414.1	WP_087713935.1	NDE08489.1	PYG01574.1
MBH74889.1	WP_008005457.1	MBU09775.1	MYF91585.1	F0TBN8
PZC42635.1	WP_124956416.1	MSS70727.1	MBS14528.1	D3E402
HCE77010.1	PSQ19454.1	MSS70727.1	MXZ10691.1	R9SJI6
MBE14505.1	NBD16670.1	Q7CRQ0	TMH88415.1	A0A166EX36
MBA03487.1	OAD18709.1	TMK21726.1	MSQ54164.1	A0A315XLL1
MBF05655.1	WP_029637489.1	MBH26744.1	WP_146883668.1	E3GZ77
MBG81921.1	WP_015129597.1	MBK50041.1	NBS65458.1	A0A328P936
MAQ48651.1	RPI91364.1	MBA26123.1	WP_015865933.1	A0A328S633
MBF50889.1	HBH60995.1	MAP36251.1	RJS83486.1	A0A2Z4L960
MBI64100.1	WP_027157527.1	MBH74206.1	MIL09188.1	A0A328S1Z8
HCI85533.1	TVR29831.1	MBL39844.1	WP_106834573.1	A0A328SNG7
MBF15406.1	PIY97183.1	MQF83190.1	WP_008942891.1	Q7BJX9
MBJ27525.1	WP_050036812.1	MBG73889.1	WP_066658568.1	Q0P8I7
MBU80259.1	WP_137197463.1	MBP38294.1	WP_076812279.1	A0A003
MYA61309.1	WP_082229590.1	MBI63864.1	PHY07089.1	D3E402
MXY46834.1	WP_074878330.1	MBI20755.1	OGA49387.1	O29886
MSR82129.1	WP_119814303.1	RKT09404.1	WP_151115507.1	A3MUJ4
MXW79300.1	WP_066384991.1	WP_067132808.1	WP_027299847.1	O58151
GBD11358.1	PSQ11290.1	WP_135141314.1	TET47504.1	Q7BJX9
HCK12766.1	WP_090309634.1	MQA86369.1	MBS11416.1	Q9WYX9
PYM11923.1	ESS03334.1	WP_141779676.1	MBK34963.1	Q8T8E9
WP_128225342.1	WP_123078245.1	WP_049743951.1	WP_006184508.1	P93031
ESS13054.1	ELY90342.1	WP_093853487.1	WP_012943574.1	Q8NBZ7
ERG96137.1	WP_142856984.1	WP_089303225.1	WP_007982414.1	

Supplementary Figures

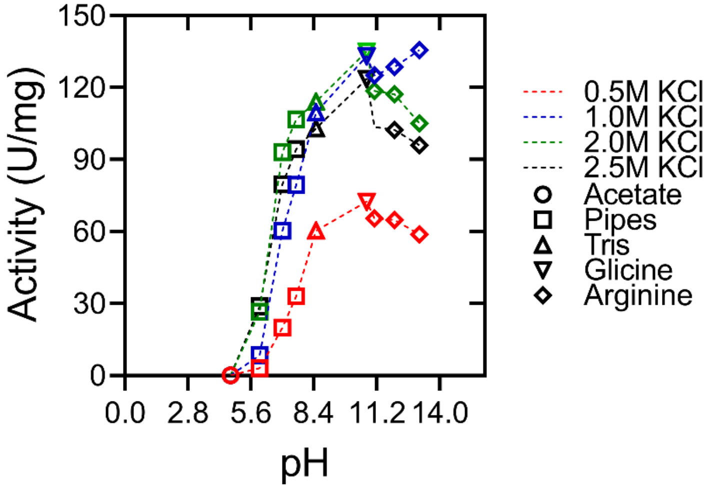

Supplementary Figure 1. Effect of pH in *Hv*G6PDH activity. The reactions were assayed at 25°C in the presence of 3 mM NAD^+ and 50 mM G6P at 100 mM of each buffer.

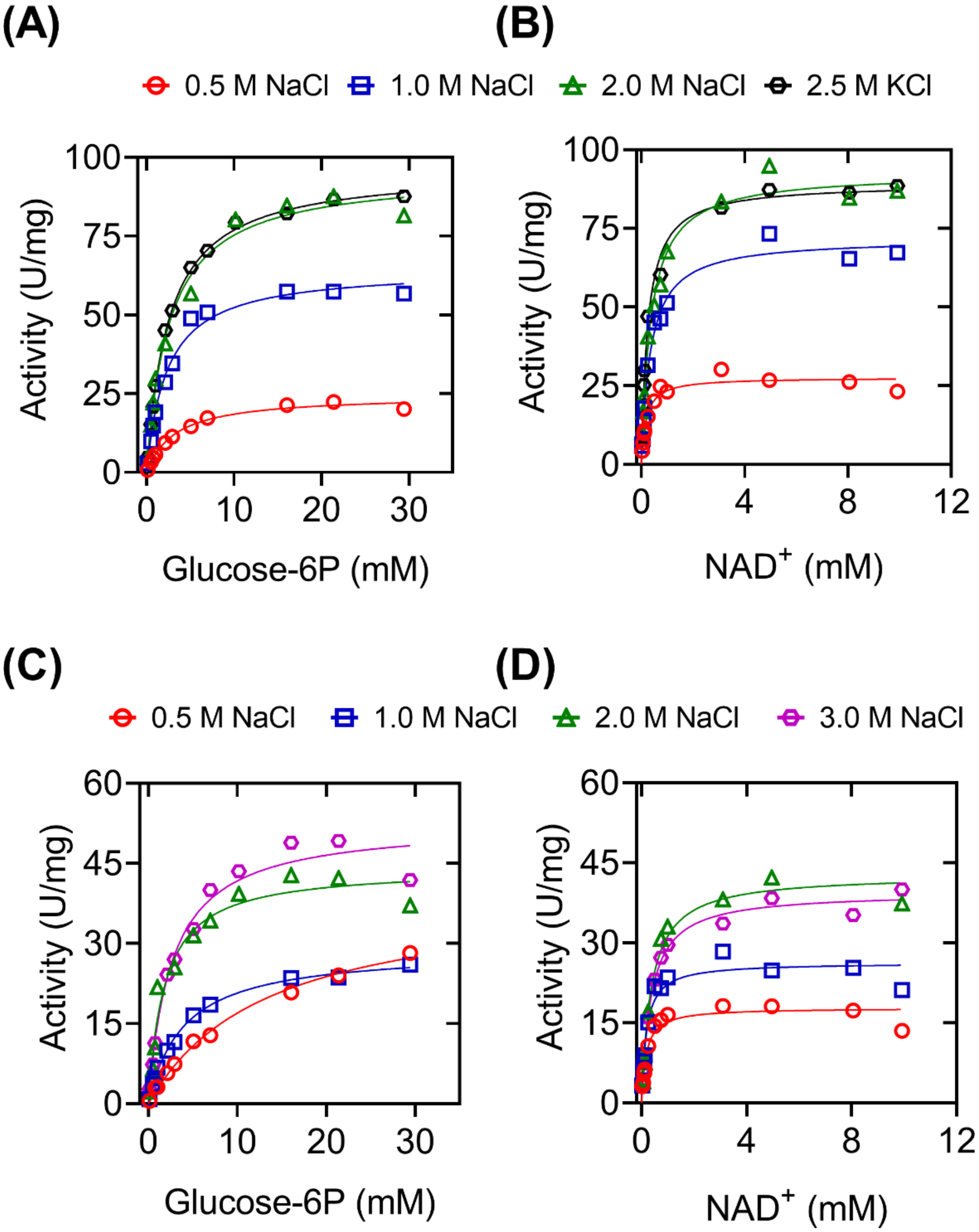

Supplementary Figure 2. Kinetic characterization of *Hv*G6PDH (A) Representative saturation curves for glucose-6-phosphate in presence of KCl. **(B)** Representative saturation curves for nicotinamide dinucleotide in presence of KCl. **(C)** Representative saturation curves for glucose-6-phosphate in presence of NaCl. **(D)** Representative saturation curves for nicotinamide dinucleotide in presence of NaCl. The reactions were assayed at 25°C, pH 7.5 and saturating concentrations of the co-substrate.

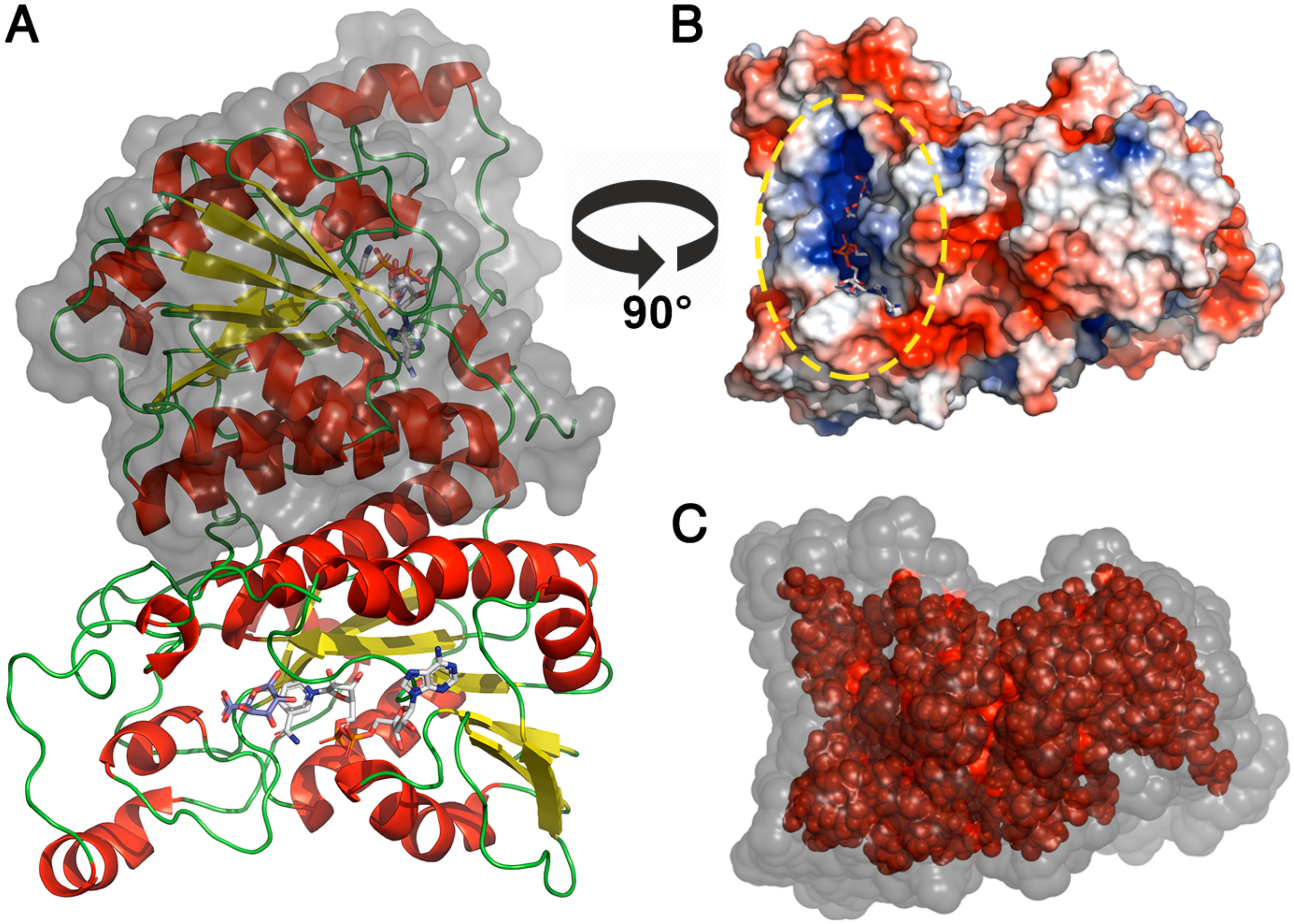

Supplementary Figure 3. Structure of the non-halophilic dimeric Uronate dehydrogenase from *Agrobacterium fabrum*. (A) Dimeric *Af*UDH architecture. Each monomer is represented as a cartoon; α helix (red), β-sheets (yellow) and loops (green). The surface of one of the monomers is shown in gray. **(B)** Surface electrostatic potential for the *Af*UDH structure, being blue for positive and red for negative potential (±3 kB T/e). Structure is represented as the solvent excluded surface (Connolly surface SES). The green oval denotes the active site. No observable predominant charge on the protein surface. **(C)** Residues from the hydrophobic core (inner shell) using as criteria less than 20 Å^2 exposed. Structure is represented as the solvent accessible surface (SAS), the surface is represented in gray and the residues from the inner shell as red spheres.

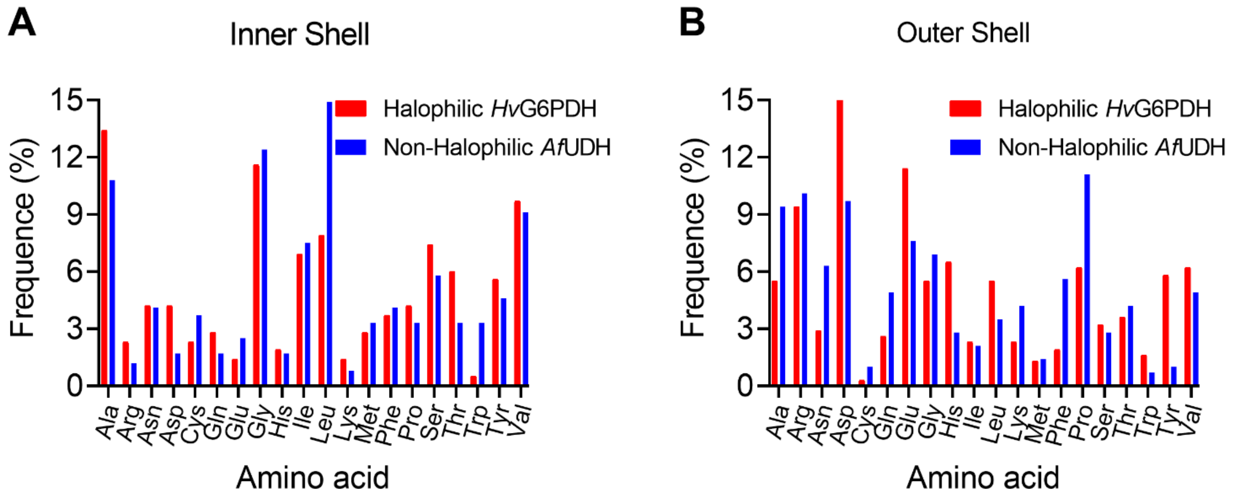

Supplementary Figure 4. Amino acid frequency distribution for halophilic *Hv*G6PDH and non-halophilic *Af*UDH. (A) Inner shell **(B)** Outer shell.

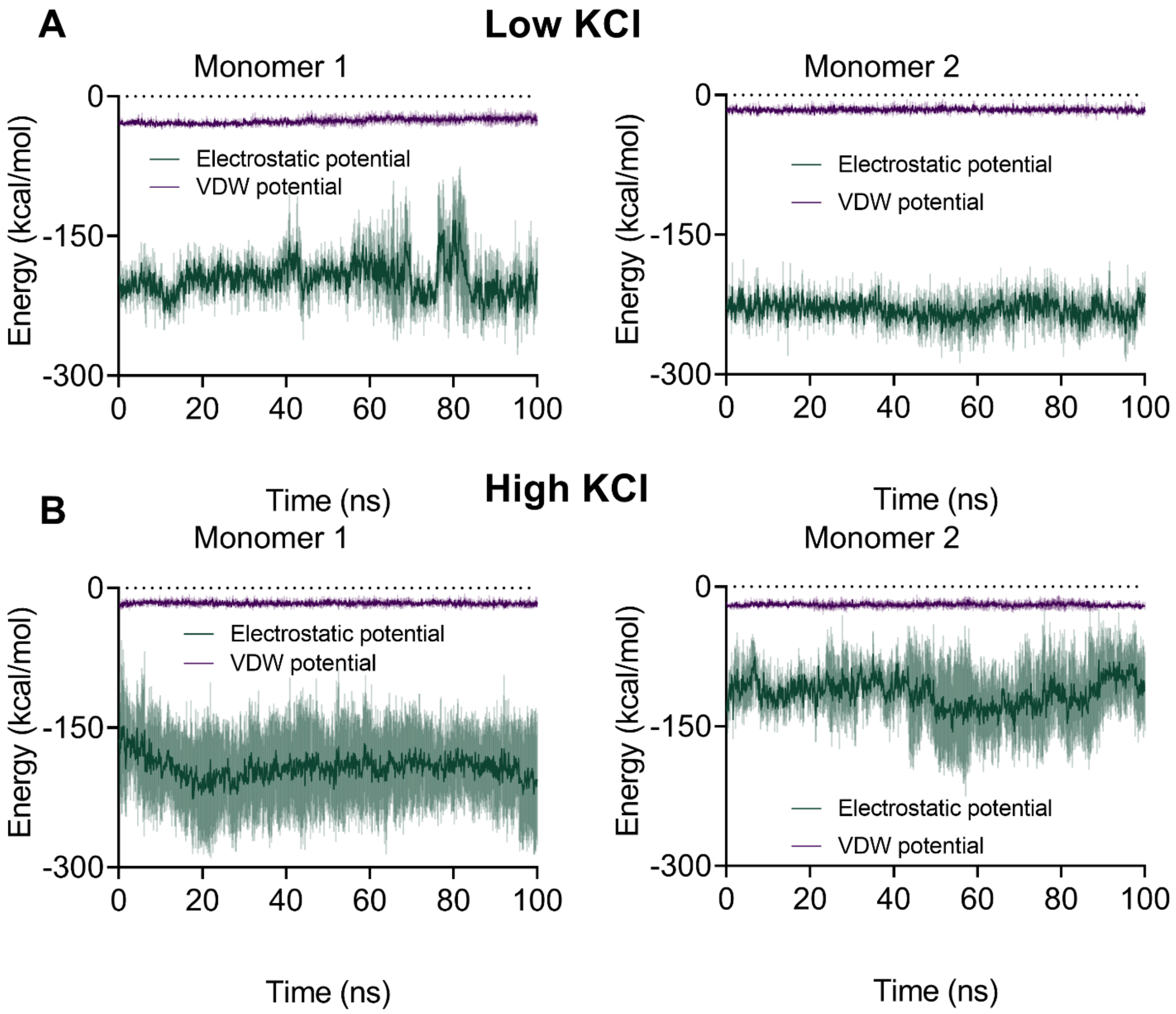

Supplementary Figure 5. Average electrostatic and van der Waals potential energies between dimeric *Hv*G6PDH and glucose-6-phosphate for each monomer at low and high salt concentration. Colored lines represent the average values of three 100ns replicates with a 1 Kcal/mol*Å^2 restriction on the ligands and to the hydroxyl group of the catalytic residue Y152, the ±SD is shown as a shaded area. **(A)** At low salt concentration the energy profile of both monomers displays an electrostatic attraction between each monomer and G6P, and a lower attractive interaction for the VDW potential. **(B)** During the molecular dynamics at high salt concentration, each monomer displays an electrostatic attraction between the protein and G6P and a lower attractive interaction for the VDW potential.

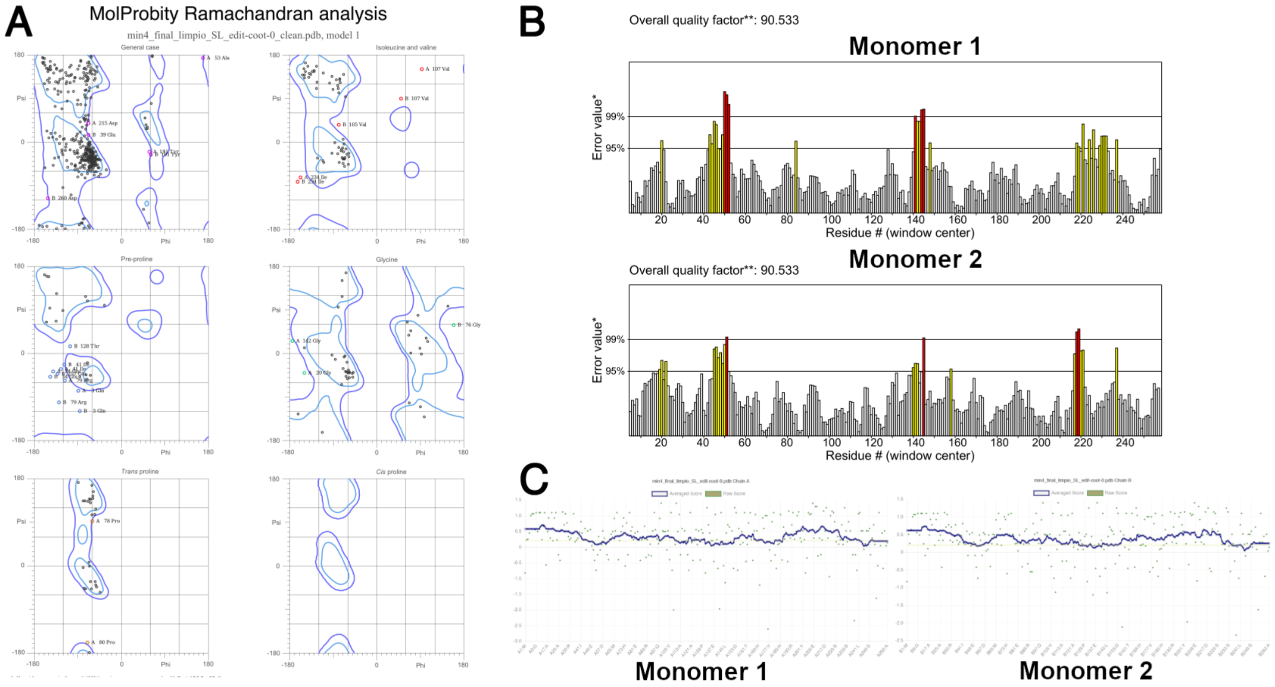

Supplementary Figure S6. Homology model validation (A) Ramachandran plot analysis **(B)** ERRAT analysis (C) Verify 3D analysis.
